# Continued Complexity of Mutations in Omicron Sublineages

**DOI:** 10.3390/biomedicines10102593

**Published:** 2022-10-16

**Authors:** Austin N. Spratt, Saathvik R. Kannan, Kalicharan Sharma, Shrikesh Sachdev, Shree L. Kandasamy, Anders Sönnerborg, Christian L. Lorson, Kamal Singh

**Affiliations:** 1 Bond Life Sciences Center, University of Missouri, Columbia, MO 65211, USA; 2 Department of Pharmacology, Delhi Pharmaceutical Sciences and Research University, New Delhi 110017, India; 3 Department of Laboratory Medicine, Division of Clinical Microbiology, Karolinska Institute, 17177 Stockholm, Sweden; 4 Department of Veterinary Pathobiology, University of Missouri, Columbia, MO 65211, USA

**Keywords:** variant of concern (VOC), Omicron sublineages, revertant mutations, structural flexibility, molecular dynamics, mutant modeling

## Abstract

The latest SARS-CoV-2 variant of concern (VOC), Omicron (B.1.1.529), has diversified into more than 300 sublineages. With an expanding number of newly emerging sublineages, the mutation profile is also becoming complicated. There exist mutually exclusive and revertant mutations in different sublineages. Omicron sublineages share some common mutations with previous VOCs (Alpha, Beta, Gamma, and Delta), indicating an evolutionary relationship between these VOCs. A diverse mutation profile at the spike–antibody interface, flexibility of the regions harboring mutations, mutation types, and coexisting mutations suggest that SARS-CoV-2’s evolution is far from over.

## 1. Introduction

Severe acute respiratory coronavirus 2 (SARS-CoV-2) has been evolving into different variants since its emergence in December 2019. To date, five official variants of concern (VOCs)—Alpha (B.1.1.7), Beta (B.1.351), Gamma (P.1), Delta (B.1.617.2), and Omicron (B.1.1.529)—have been reported. The latest VOC, Omicron (B.1.1.529), was reported in late November 2021 [1]. It has diversified into more than 300 sublineages to date, and new sublineages continue to emerge. The ultimate sources and the timing of the evolution of these variants remain largely unknown [2,3]. In fact, some sublineages—such as BA.1, BA.1.1, BA.2, and BA.3—appear to have evolved around the same time [3,4,5] or even before the discovery of Omicron itself.

Several factors, including persistent viral infection in immunocompromised individuals, alternate hosts, pressure of antibodies induced by prior infections or vaccination, and the function of restrictive factors such as APOBEC (apolipoprotein B mRNA editing enzyme, catalytic polypeptide) and ADARs (adenosine deaminases acting on RNAs), can lead to the acquisition of new mutations [6,7,8], which can potentially weaken the immune-mediated neutralization [9,10,11,12]. It remains unclear whether any of these factors contributed to the evolution of Omicron.

## 2. Materials and Methods

### 2.1. Flowchart of the Study

**Figure 1:**
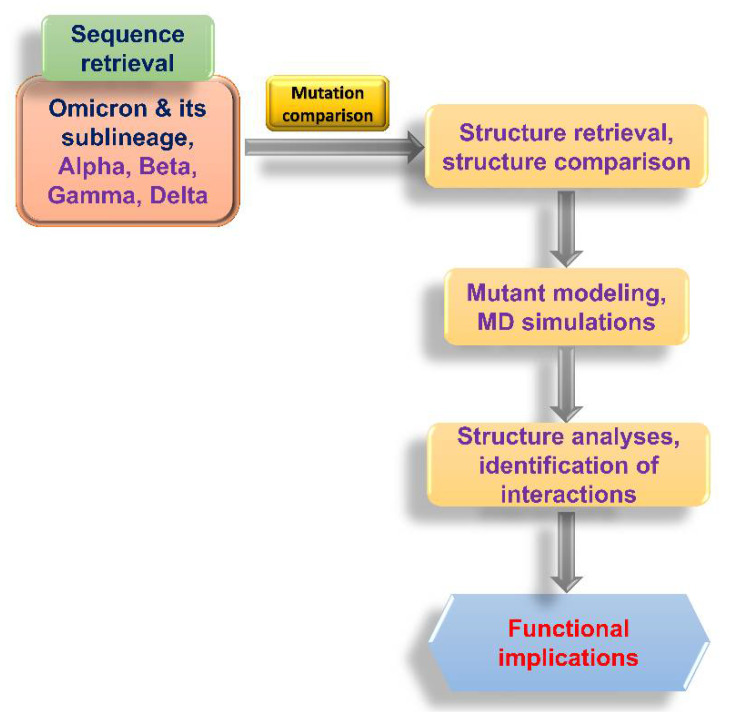
Flowchart of the analyses conducted in this study.

A flowchart of the analyses conducted in this study is shown in Figure 1. Briefly, the sequences of Omicron and its sublineages, along with the Alpha, Beta, Gamma, and Delta VOCs, were obtained from GISAID [13]. The mutations were then identified using an in-house R script that was based on the API provided by outbreak.info [14]. Where available, the structures of the spike receptor-binding domain (S-RBD/ACE2 or S-RBD/monoclonal antibody (mAb) were retrieved from the Protein Data Bank (www.rcsb.org, accessed on 6 August 2022). Structures were aligned using the structure of the S-RBD/ACE2 complex reported by Lan et al. [15], using the ‘align’ function of PyMOL (Schrodinger LLC, New York, NY, USA). Mutant modeling and molecular dynamics simulations were conducted as described in the following sections. The interactions between residues were determined using either PyMOL or Maestro (Schrodinger LLC, New York, NY, USA). Functional implications were drawn from the structure/sequence analyses.

### 2.2. Sequence Acquisition and Analysis

The prevalence of each mutation in BA.1, BA.1.1, BA.2, BA.2.1.1, BA.3, BA.3.1, BA.4, and BA.5 was calculated using an R script that was based on the API (application programming interface) available at outbreak.info. This API has an interface with the GISAID repository [13] of SARS-CoV-2 sequences. The modified R script rearranges the occurrence of the order of Omicron sublineages or other VOCs. The details of the API can be found on the GitHub page https://github.com/outbreak-info/R-outbreak-info accessed on 6 August 2022.

### 2.3. Structural Analysis

The structures of the S-RBD/ACE2 and S-RBD/mAb complexes were retrieved from the Protein Data Bank (www.rcsb.org, accessed on 6 August 2022). Mutations at specific positions were generated using the ‘Mutagenesis’ wizard of PyMOL (Schrodinger LLC, New York, NY, USA). The structures were aligned using the ‘align’ tool of PyMOL. All distance measurements were carried out in PyMOL. The interface residues were identified using the R package Bio3D [16]. The chord diagram was generated using an in-house R script. All scripts used here are available upon request after fulfilment of requirements by the University of Missouri.

### 2.4. Molecular Dynamics (MD) Simulations

For MD simulations, we used the crystal structure of the spike receptor-binding domain (S-RBD) in complex with human angiotensin-converting enzyme 2 (ACE2) (PDB file 6M0J) [15]. All MD simulations were conducted in TIP3P [17], water-filled, truncated, isometric, octahedral periodic boxes of at least 12 Å depth on all sides of the protein surface. Ten Na^+^ and ten Cl¯ ions were used to neutralize the total charge on the S-RBD/ACE2 complex and to maintain the ionic strength of 145 mM. Monovalent (Na^+^ and Cl¯) ion positions were randomized at 5.0 Å from other solute atoms and 3.0 Å from one another using different seeds to generate five model replicates. All MD simulations were run at 300 K with a pressure of 1.013 bar for 60 ns. The MD trajectories were analyzed and generated by VMD [18].

## 3. Results

**Figure 2:**
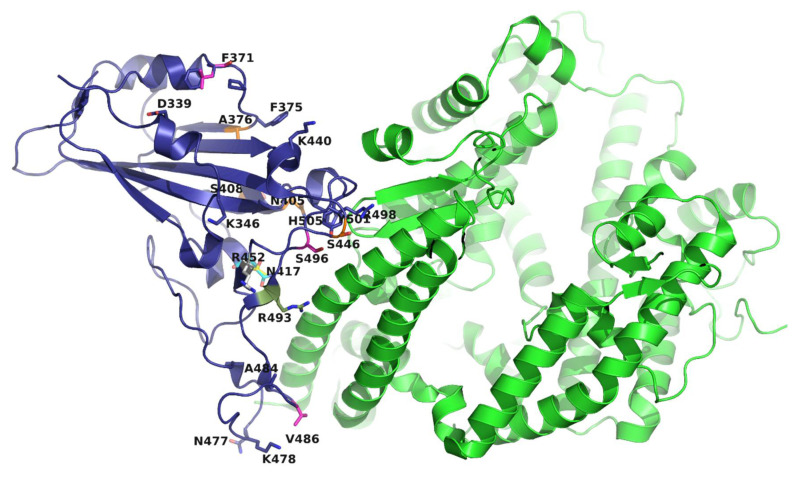
Mutations in the S-RBD of Omicron and its sublineages (see Figure 3a). The S-RBD (purple) and ACE2 (green) are rendered as ribbons. Mutations (shown as sticks) in 8 or more sublineages are colored purple; mutation in one sublineage is colored yellow; mutations in 2, 3, 4, 5, and 6 sublineages are colored magenta, gray, orange, cyan, and forest green, respectively. This figure was generated using the PDB file 6M0J [15].

The Omicron sublineages have: (i) a high number of mutations [19,20] and (ii) a remarkably diverse mutation profile [20]. Of the total 44 mutations in the S protein, 22 are in the S-RBD (Table 1). Of these, 16 mutations are at the S-RBD–ACE2 interface (Figure 2). A comparison of mutations in the spike protein, as of 12 June 2022 (Figure 2), highlights the differences in the mutation profiles of a few select Omicron sublineages. Thus, the low-prevalence mutations T19I, L24S, and DEL25/27 in BA.1-related sublineages are present at high prevalence in BA.2, BA.4, and BA.5 (Figure 2). Conversely, the highly prevalent mutations A67V, T95I, and DEL143/145 of BA.1 are absent in BA.2, BA.4, and BA.5. Moreover, two mutations in BA.1 and BA.3 (i.e., G446S and G496S) reverted back to the wild-type (WT, Wuhan-Hu-1) residues (G446 and G496) in BA.2, BA.4, and BA.5 (Figure 2). Other notable differences include inter-sublineage mutation patterns (discussed below).

**Figure 3:**
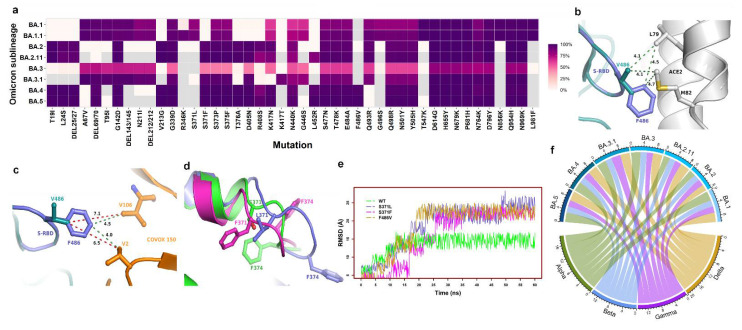
Prevalence of mutations in Omicron sublineages and their structural impacts: (**a**) An example of the mutation patterns in different Omicron sublineages. Sublineages BA.1.1 and BA.2.11 are included here to show that there exist inter-sublineage differences in the mutations. Sublineage B.1.1 has an R346K mutation that is not present in B.1 and Omicron (B.1.1.529). Similarly, BA.2.11 has an L452R mutation that is not present in BA.2. (**b**) The location and interactions of F486/V486 with ACE2. This figure was generated from PDB entry 7ZF7 [21]. The WT (Wuhan-Hu-1) S-RBD is shown as violet ribbons, whereas ACE2 is colored grey. The amino acid residues are rendered as ball-and-stick models. The carbon atoms in this and subsequent panels are colored in the same colors as the ribbons, whereas sulfur atoms are shown in gold and nitrogen in blue. V486-containing S-RBDs (as in BA.4 and BA.5) are shown in teal, as are the carbon atoms. The green dotted lines represent the distance between the F486/V486 and ACE2 residues. (**c**) The impact of the F486V mutation on mAb binding. This figure was generated from PDB entry 7ZF8 [21], representing the binding of the BA.2 S-RBD with the COVOX 150 antibody. The V486 mutation is shown in teal and the amino acid residues of COVOX 150 are shown in orange. (**d**) The change in the conformation of the loop containing S371 in WT (green), L371 in BA.1 (deep blue), and F371 in BA.2 (magenta). It is clear from the position of F374 (located on the same loop) that the loop conformation is altered significantly, depending upon the omicron sublineage. (**e**) The trajectory of the root-mean-square deviation of S-RBD Cα-atoms in WT, S371L, S371F, and F486V over 60 ns MD simulations. (**f**) A chord diagram shows common mutations between selected Omicron sublineages and the Alpha, Beta, Gamma, and Delta VOCs. The thickness of the end of the chord represents the number of mutations (two or three).

The mutations K417N and K417T are signature mutations of Beta and Gamma, respectively. The majority of Omicron sublineages have K417N. However, BA.2.18, BA.2.38, BA.2.40.1, and BA.3.1 have K417T [14]. Intriguingly, K417N and K417T are mutually exclusive, i.e., the sublineages harboring K417T do not have K417N, and vice versa [14]. L452R, a signature mutation of Delta, is highly prevalent (~90%) in BA.2.11, BA.4, and BA.5, but not in BA.2 (Figure 3a). Except for Q493R, all BA.2 mutations are present in BA.4 and BA.5 (Figure 3a), but BA.4 and BA.5 have an additional three mutations: Δ69/70, L452R, and F486V.

Another mutation, F486V, is only prevalent in BA.4 and BA.5 (Figure 3a). F486V confers the loss of potency for multiple neutralizing antibodies [22]. F486 is located on a loop and forms hydrophobic interactions with L79 and M82 of ACE2 (Figure 3b). A simple mutant modeling helped to predict the mechanisms involved in maintaining the hydrophobic interaction of V486-containing S proteins with ACE2, as well as antibody evasion. The structures of the F486V spike/ACE2 or spike receptor-binding domain (S-RBD)/ACE2 complexes and any mAbs are not known. Hence, the crystal structures of the BA.2 S-RBD/ACE2 complex (PDB entry 7ZF7) and the BA.2-RBD/COVOX 150 Fab complex (PDB entry 7ZF8) [21] were used to generate the F486V mutation with the ‘Prime’ software of Schrodinger Suite (Schrodinger LLC, New York, NY, USA) (Figure 3b,c). The mutant modeling suggests that F486V retains the same interaction distance to L79 and M82 (both on ACE2) (green dotted lines) (Figure 3b). However, the hydrophobic interactions between F486 (S-RBD of BA.2) and V2 and V106 (of COVOC 150) are lost upon acquisition of the F486V (BA.4 and BA.5) mutation, as the distances from V486 to V106 and V2 (7.1 Å and 6.5 Å, respectively) (red dotted lines) are significantly greater than the distances from F486 to V106 and V2 (4.5 Å and 4.0, respectively) (green dotted lines) (Figure 3c).

Another intriguing factor associated with F486V is that the sublineages containing F486V do not have the Q493R mutation. Instead, they have the revertant mutant Q493 (as in the WT), suggesting that if there was any reduction in S-RBD–ACE2 binding, the revertant mutation would functionally compensate by selecting Q493 (WT) in place of R493 (as in BA.1, BA.2, BA.3, and related sublineages). The loop harboring F486 also has another Omicron signature mutation (E484A), which abrogates the binding of the LY-CoV555 monoclonal antibody (mAb) to S-RBD [23]. It appears that BA.4, and BA.5 evolved with the additional F486A mutation to evade not only LY-CoV555, but also other antibodies (such as COVOC 150) that would have neutralizing activity. It is also possible that BA.4 and BA.5 evolved with the F486V mutation in conjunction with E484A to adopt a flexible loop conformation that would be capable of maintaining hydrophobic interactions but reduce the binding to antibodies [24] (Figure 3a,b). The conformational flexibility of the loops containing mutations that may escape antibody binding is evident from the comparison of the S-RBD structures of different VOCs. The loops harboring S371 (WT), S371L (in BA.1), and S371F (in BA.2) have strikingly different conformations (Figure 3d), as do S-RBDs bound to the S304 mAb [23]. To test whether the S371L/F mutation influences the S-RBD’s conformational flexibility, we conducted molecular dynamics (MD) simulations. The MD simulations (Figure 3d) showed that WT stabilizes by ~12 ns, whereas the S371L and S371F mutations take ~22 ns and ~25 ns, respectively, and the Cα fluctuations of S371L/F remain greater than those of WT throughout the simulation. F486V was also included in the MD simulations to examine its impact. In line with our predictions, the MD results demonstrated that the F486V mutation also increases the flexibility of the S-RBD compared to WT (Figure 3d).

Deletion mutations DEL69/70 and L452R are signature mutations of Alpha and Delta, respectively. DEL69/70 is highly prevalent in all sublineages, except for BA.2 (Figure 3a). DEL69/70 itself does not evade antibody binding, but increases infectivity [25]. L452R evades cellular immunity and increases infectivity [26,27], and the L452R mutation in Omicron greatly enhances its ability to infect the lung tissues of humanized ACE2 mice [27]. These examples of mutation profile differences in Omicron sublineages and phenotypes associated with these mutations suggest that different Omicron sublineages may have different pathogenicity.

Comparison of ~10 million sequences (outbreak.info) shows that 2–3 mutations are common between Omicron and Alpha, Beta, Gamma, and Delta (Figure 3e). The chord diagram (Figure 3e) shows that BA.1 shares three common mutations with Delta and Alpha, and two with Beta and Gamma. Sublineages BA.2.11 and BA.3.1 were included to demonstrate inter-sublineage differences. For example, BA.2.11 has three common mutations with Delta, whereas BA.2 has only two. Similarly, BA.3.1 (the only reported BA.3-related sublineage) shares three mutations with Gamma, whereas BA.3 has only two. The VOCs Alpha, Beta, Gamma, and Delta have 9, 9, 12, and 10 mutations, respectively. The presence of only 2–3 common mutations between Omicron sublineages and the previous four VOCs, with the remainder (6–9) being the same as in the WT, suggests that the Omicron sublineages most likely evolved independently from the WT virus and had time to diversify before they were discovered [3].

## 4. Conclusions

In summary, the analyses presented above show that the Omicron sublineages have a complex mutation profile, including mutually exclusive (i.e., K417N and K417T) and revertant mutations (i.e., G446 and G496). The structural flexibility, types of mutations—such as S371L (in BA.1) and S371F (in BA.2)—and the difference in mutation profile at the interface of mAb binding, add additional complexity to the evolution of Omicron and its sublineages. Furthermore, the emergence of recombinant Omicron viruses such as XD, XE, XF, XL, XN, XP, XQ, XU, and XV indicates that more Omicron sublineages will emerge in the future. In fact, the number of Omicron sublineages is increasing at an unprecedented rate, further confirming the complex evolutionary characteristics of this variant. As of 3 August 2022, there were over 271 sublineages of Omicron, while there were only ~75 on 1 May 2022. As of 28 September 2022, there were ~325 omicron sublineages. The complex pattern of mutations in Omicron sublineages suggests increased resistance to various antibodies. New variants or sublineages of existing VOCs may also emerge under a variety of physiological and therapeutic pressures. The waning of natural and hybrid immunity to SARS-CoV-2 [28] indicates that COVID-19 is likely to exist for a long period of time. In conclusion, it is possible that COVID-19 may be heading towards endemicity, but the continued evolution of SARS-CoV-2 does not appear to be ending anytime soon.

## Figures and Tables

**Table 1:** Mutations of the S-RBD in different Omicron sublineages.

Mutation	Omicron Sublineage						
G339D	BA.1	BA.1.1	BA.2	BA.2.11	BA.3	BA.4	BA.4
R346K		BA.1.1					
S371L	BA.1	BA.1.1					
S371F			BA.2	BA.2.11	BA.3	BA.4	BA.4
S373P	BA.1	BA.1.1	BA.2	BA.2.11	BA.3	BA.4	BA.4
S375F	BA.1	BA.1.1	BA.2	BA.2.11	BA.3	BA.4	BA.4
T376A			BA.2	BA.2.11		BA.4	BA.4
D405N			BA.2	BA.2.11	BA.3	BA.4	BA.4
R408S			BA.2	BA.2.11		BA.4	BA.4
K417N	BA.1	BA.1.1	BA.2	BA.2.11	BA.3	BA.4	BA.4
N440K	BA.1	BA.1.1	BA.2	BA.2.11	BA.3	BA.4	BA.4
G446S	BA.1	BA.1.1			BA.3		
L452R				BA.2.11		BA.4	BA.4
S477N	BA.1	BA.1.1	BA.2	BA.2.11	BA.3	BA.4	BA.4
T478K	BA.1	BA.1.1	BA.2	BA.2.11	BA.3	BA.4	BA.4
E484A	BA.1	BA.1.1	BA.2	BA.2.11	BA.3	BA.4	BA.4
F486V						BA.4	BA.4
Q493R	BA.1	BA.1.1	BA.2	BA.2.11	BA.3		
G496S	BA.1	BA.1.1					
Q498R	BA.1	BA.1.1	BA.2	BA.2.11	BA.3	BA.4	BA.4
N501Y	BA.1	BA.1.1	BA.2	BA.2.11	BA.3	BA.4	BA.4
Y505H	BA.1	BA.1.1	BA.2	BA.2.11	BA.3	BA.4	BA.4

## Data Availability

Outbreak.info and GISAID (https://www.gisaid.org).
